# Testosterone Is Inversely Related to Brain Activity during Emotional Inhibition in Schizophrenia

**DOI:** 10.1371/journal.pone.0077496

**Published:** 2013-10-31

**Authors:** Ans Vercammen, Ashley J. Skilleter, Rhoshel Lenroot, Stanley V. Catts, Cynthia Shannon Weickert, Thomas W. Weickert

**Affiliations:** 1 Neuroscience Research Australia, Randwick, NSW, Australia; 2 Schizophrenia Research Institute, Darlinghurst, NSW, Australia; 3 School of Psychiatry, University of New South Wales, Randwick, NSW, Australia; 4 School of Medicine, University of Queensland, Brisbane, QLD, Australia; United Graduate School of Child Development, Osaka University, Japan

## Abstract

Sex steroids affect cognitive function as well as emotion processing and regulation. They may also play a role in the pathophysiology of schizophrenia. However, the effects of sex steroids on cognition and emotion-related brain activation in schizophrenia are poorly understood. Our aim was to determine the extent to which circulating testosterone relates to brain activation in men with schizophrenia compared to healthy men during cognitive-emotional processing. We assessed brain activation in 18 men with schizophrenia and 22 age-matched healthy men during an emotional go/no-go task using fMRI and measured total serum testosterone levels on the same morning. We performed an ROI analysis to assess the relationship between serum testosterone and brain activation, focusing on cortical regions involved the emotional go/no-go task. Slower RT and reduced accuracy was observed when participants responded to neutral stimuli, while inhibiting responses to negative stimuli. Healthy men showed a robust increase in activation of the middle frontal gyrus when inhibiting responses to negative stimuli, but there was no significant association between activation and serum testosterone level in healthy men. Men with schizophrenia showed a less pronounced increase in activation when inhibiting responses to negative stimuli; however, they did show a strong inverse association between serum testosterone level and activation of the bilateral middle frontal gyrus and left insula. Additionally, increased accuracy during inhibition of response to negative words was associated with both higher serum testosterone levels and decreased activation of the middle frontal gyrus in men with schizophrenia only. We conclude that endogenous hormone levels, even within the normal range, may play an enhanced modulatory role in determining the neural and behavioural response during cognitive-emotional processing in schizophrenia.

## Introduction

Many observations support the relationship between sex steroid hormones and the development and course of schizophrenia. Sex differences have been demonstrated in the onset and severity of schizophrenia with men being more severely affected [1]. Estrogen may act as a neuroprotective factor in women with schizophrenia [2], but little is known about the role of testosterone in men with schizophrenia. The peak in age-of-onset during adolescence suggests a link between exposure to increased androgens and development of schizophrenia in at-risk men. However, a number of studies, including first episode and ultra-high risk, have reported either low or normal circulating testosterone levels in men with schizophrenia [3]–[6]. The evidence for a relationship with testosterone is strengthened by reports of increased negative symptoms and worse cognitive function in association with low endogenous testosterone levels in men with schizophrenia [7]–[11]. Furthermore, exogenous testosterone supplementation can reduce negative symptoms in men with schizophrenia [12].

Variation in testosterone level has been shown to influence aspects of affective [13] and social behaviour [14]–[16], as well as cognitive performance in healthy men [17], and those with schizophrenia [10]. Evidence from neuroimaging studies in healthy adults indicates that fronto-limbic activation correlates with testosterone level during emotion-related processing [18]–[21]. However, little is known about the relationship between endogenous testosterone levels and brain activation associated with cognitive or affective processes in schizophrenia. One recent study assessing brain activation associated with visual-spatial ability reported a positive correlation with testosterone in healthy men but no correlation in men with schizophrenia [22]. However, this study also reported lower testosterone levels in the patient group. We predict that testosterone levels may affect other neurocognitive processes that are more central to the illness, such as executive control, attention [23] and emotion processing and regulation [24].

The aim of the current study was to test whether circulating testosterone levels were correlated with brain activation during cognitive-emotional processing in men with schizophrenia. We used an emotional go/no-go paradigm, which activates dorsal prefrontal executive control brain regions in addition to insular and limbic cortex associated with emotion regulation [25], [26]. Brain activation during this task was previously shown to be sensitive to sex steroid modulation of prefrontal and cingulate activity in healthy adults [27]. We focused on negative valence, since negative emotion has been shown to have stronger interference effects on cognitive processes in schizophrenia [28] and because our own work indicated that positive emotional go/no-go conditions did not engage the same frontal network as robustly [26].

Based on existing evidence of a relationship between endogenous testosterone and brain activity during emotion-related tests, as well as testosterone’s putative involvement in the pathophysiology and course of schizophrenia, we predicted that the relationship between circulating testosterone and task-related brain activation during an emotional go/no-go task would differ between men with schizophrenia and healthy men. We tested this hypothesis in an a priori-defined cortical network known to be involved in controlling and integrating cognitive and emotional processing.

## Materials and Methods

### Ethics Statement

All procedures were approved by the University of New South Wales and the South Eastern Sydney and Illawarra Area Health Service Human Research Ethic Committees and the study was conducted according to the principles expressed in the Declaration of Helsinki. Written informed consent was obtained from all participants prior to entry into the study. Ability to provide informed consent was assessed first by the participant’s referring clinician who was not associated with the study and an additional study physician prior to inclusion in the study. None of the participants had significant cognitive impairment (FSIQ<75) which would interfere with their ability to provide informed consent. All potential participants who declined to participate or otherwise did not participate were eligible for treatment and were not disadvantaged in any other way by not participating in the study.

### Participants

Eighteen right-handed men with schizophrenia or schizoaffective disorder and twenty-two right-handed healthy men took part in the fMRI experiment. Patients were recruited via information leaflets distributed within the local area health service and through a national television documentary. Healthy men were recruited via advertisements in newspapers and on community notice boards. Data from a subset of these participants (15 men with schizophrenia and 11 healthy men) were included in a previous report from our lab [26]. General exclusion criteria for the study were the presence of a concurrent Axis I diagnosis other than schizophrenia/schizoaffective disorder for the patient group and a personal history of psychiatric illness or a first degree relative with schizophrenia/schizoaffective disorder for the healthy control group. For both participant groups further exclusion criteria were a history of recent (within 5 years) substance abuse or dependency, uncontrolled diabetes or cardiovascular disease including hypertension, a history of neurological disorders, head injury with loss of consciousness, epileptic seizures, and structural brain abnormalities. All men with schizophrenia received out-patient care and had been receiving treatment with antipsychotics for at least one year prior to inclusion in the study. Psychiatric diagnosis was obtained from medical record review and confirmed by means of the Structured Clinical Interview for DSM-IV-TR, (SCID) [29]. Current symptom severity was assessed with the Positive and Negative Syndrome Scale (PANSS) [30]. A trained psychologist or psychiatrist conducted all clinical and diagnostic assessments. All participants were assessed with a four subtest version of the Wechsler Adult Intelligence Scale, 3rd edition (WAIS-III) [31] to estimate current intellectual functioning.

### Hormonal Assays

Fasting peripheral blood was obtained by venepuncture between 9 and 11 am to control for circadian variation in hormone levels. Immediately following collection, clotted and heparinised blood samples were delivered on ice to the Prince of Wales Hospital South Eastern Area Laboratory Services Pathology unit. Sex steroid levels were measured from serum and provided a measure of “total” circulating hormone levels. Testosterone and estradiol were assayed using a solid-phase competitive chemiluminescent immunometric assay and prolactin was assayed using a solid-phase two-site chemiluminescent immunometric assay (Siemens Healthcare Diagnostics Products Ltd.). Reference ranges were set at 5.6–23.6 nmol/L for testosterone and 0–206 pmol/L for estradiol, sensitivity of the assay was 0.7 nmol/L for testosterone and 73 pmol/L for estradiol and the interassay coefficients of variation (CV) were ≈9.7% and ≈13.5% for testosterone and estradiol, respectively. For prolactin, reference ranges were set at 0–372 mlU/L, sensitivity of the assay was 11.0 mlU/L, the intra-assay CV was 6 and the interassay CV was ≈6.5%.

### Emotional Go/no-go Task

The stimuli used in the emotional go/no-go task were selected from the Affective Norms for English Words (ANEW) [32] stimulus set, which provides normative valence and arousal ratings. We selected 40 neutral, 20 positive and 20 negative words from the normative lists and matched them for word length and frequency. Positive and negative words also matched for arousal. All participants rated the stimuli in a pen-and-paper forced choice questionnaire, indicating for each of the stimuli whether they were perceived as positive, neutral or negative. These subjective ratings were used to define response accuracy in the emotional go/no-go task on an individual basis.

All participants received detailed verbal instructions prior to scanning. The emotional go/no-go task includes four conditions: 1) inhibit neutral/respond to negative: responding to negative words while inhibiting responses to neutral ones and 2) inhibit negative/respond to neutral: responding to neutral words while inhibiting responses to negative ones. The two remaining task conditions involve responding to/inhibiting responses to positive words compared to neutral words. For the purposes of the current study, we selectively focused on the task conditions with negative valence, i.e., conditions 1 and 2 above, based on previous work showing that these conditions produced robust activation of a prefrontal network, whereas contrasting positive and neutral words elicited much weaker activation [26].

Participants were instructed to respond as quickly as possible to stimuli of the specified valence (targets), but to ignore stimuli of other valences (distracters). A single trial consisted of a fixation cross for 600 ms, a word stimulus presented in the center of the screen for 600 ms, and an inter-stimulus response interval of 1800 ms. Stimuli were presented in blocks of 10 trials. Each block was preceded by an instruction: the word “NEGATIVE,” “POSITIVE” or “NEUTRAL” appeared in capital letters to indicate the target valence. There were 4 different blocks with 5 negative and 5 neutral stimuli. Each of these blocks was presented twice, once with a “NEGATIVE” instruction and once with a “NEUTRAL” instruction. The two conditions were thus identical with respect to stimuli and differed only in terms of the instruction given at the start of each block. Block order was pseudo-randomized such that the same condition could not appear in consecutive blocks. A 30 second fixation crosshair was presented at the initiation and conclusion of the scan session. The total duration was 10 minutes. Stimuli were presented via a computer screen that participants viewed via a mirror and responses were recorded via a fiber optic response pad (Lumina Systems), which collected accuracy (percent correct) and reaction time (RT) in ms.

### fMRI Scanning Procedure

Magnetic resonance imaging was performed using a 3 Tesla Phillips Achieva MRI scanner with an 8 channel bird-cage type head coil at Neuroscience Research Australia, Randwick, NSW, Australia. A T1-weighted high-resolution anatomical scan was obtained for each participant for registration purposes and to screen for anatomical abnormalities (TR, 5.4 ms; TE, 2.4 ms; FOV, 256 mm; matrix, 256×256; sagittal plane; slice thickness, 1 mm, no gap; 180 slices). Functional T2* weighted images were obtained using a gradient echo-planar imaging sequence, TR/TE = 3000/30; 32 interleaved slices, covering the whole brain, thickness = 3 mm, gap = 1 mm; voxel size 3×3×3 mm; scan repetitions = 212; flip angle = 90°; field of view = 24 cm.

### Data Analysis

#### Hormone assays

Independent sample t-tests were performed to assess group differences in testosterone, estradiol and prolactin levels. Pearson product-moment correlation coefficients were obtained among hormone levels, demographic and clinical characteristics of the sample, including age, medication dose and PANSS symptom scores.

#### Emotional go/no-go task performance

Task performance measures included reaction times (RTs) for correct responses, response accuracy (% correct responses), the number of false alarm errors and omissions. Correct responses were defined as a button press to a target and the withholding of a button press to a distracter. False alarms were defined as a button press to a distracter, whereas omissions refer to the withholding of a button press to a target stimulus. Mixed ANOVAs were performed with group as between-subjects and condition as within-subjects factors. In addition, Pearson product-moment correlation coefficients were obtained between hormone levels and task performance.

#### fMRI analysis

All analyses were performed with SPM8 (Wellcome Trust Centre for Neuroimaging), running under Matlab2010b. Four dummy scans were obtained before each fMRI data acquisition to allow for the equilibration of the MRI signal. Functional images were realigned to the first image in the time series and co-registered with the anatomical image. The images were manually reoriented to optimize the normalization process and normalized to the Montreal Neurological Institute (MNI) anatomical template using a non-linear 12 parameter affine transformation. Images were smoothed with a 10 mm FWHM Gaussian Kernel. All data sets were screened for movement artifacts (subjects were excluded if movement parameters exceeded 3 mm translation along x, y or z axes or 3 degrees of rotation) and successful normalization. To further control for motion effects, the motion parameters were also included as regressors in the first level analysis. At the first level of analysis, contrasts were created for each subject to assess the magnitude of difference in blood oxygen level-dependent (BOLD) signal between conditions of interest: inhibit negative/respond to neutral versus inhibit neutral/respond to negative, to arrive at the relative activation during inhibition of responses to negative versus neutral stimuli. At the second level, we first constructed single sample T-test models for the group of healthy men and the group of men with schizophrenia separately to assess the main task effect for the contrast of interest (*inhibit negative versus neutral*). Next, to assess the relationship between serum testosterone and BOLD response, the first level contrasts (*inhibit negative versus neutral*) were entered into a regression model with testosterone levels as a continuous predictor, for the group of healthy men and men with schizophrenia separately. In addition, we performed a control analysis to examine the relationship between testosterone and BOLD response when controlling for the level of estradiol, as the two hormones may interact.

The *inhibit negative versus neutral* contrast compares conditions that differ only in the subjective valence of the stimuli to which responses are inhibited, producing relatively subtle effects when tested at the whole brain level [26]. In order to maximize our statistical power, we confined the analyses to a set of regions of interest (ROIs), based on previous results comparing the same task conditions (*inhibit negative versus neutral*) [26]. The selected regions are known to be involved in emotional go/no-go conditions [25], [33]. The following bilateral structural ROIs were selected from the Anatomical Automatic Labeling (AAL) Atlas [34]: middle frontal gyrus, posterior cingulate, precuneus and insula. In addition, we selected a control region, the postcentral gyrus, which was not previously associated with the emotional inhibition task and for which we expected no testosterone effects [25], [27]. A structural ROI analysis was performed using the MarsBar toolbox implemented in SPM [35]. All voxels within the ROIs are treated as many samples of the same signal and a summary value, based on the mean, is calculated across all voxels in the ROI. Results are reported with FDR corrections for multiple comparisons, unless stated otherwise. Parameter estimates for the *inhibit negative versus neutral* task contrast were extracted for each of the ROIs and Pearson product-moment correlations were determined between BOLD response, task performance (accuracy), medication dosage, and symptom severity (PANSS scores).

## Results

### Demographic, Clinical and Neuropsychological Characteristics

Table 1 summarizes the demographic, clinical and neuropsychological characteristics of the sample. Statistical comparison using t-tests confirmed that the groups were well matched on age. As expected, healthy men had a significantly higher estimated full scale IQ and education level compared to the men with schizophrenia. PANSS scores for the men with schizophrenia were within the mild to moderately severe range.

**Table 1:** Demographic and clinical characteristics of the sample, comprising 18 males with schizophrenia and 22 healthy males.

	Males with schizophrenia		Healthy males		Statistic
	Mean (SD)	Range	Mean (SD)	Range	
**AGE (years)**	34.9 (9.1)	25–50	32.3 (8.6)	22–48	t(38) = 0.7; p>.10
**EDUCATION LEVEL (years)**	13.3 (2.7)	10–19	14.9 (2.2)	10–18	t(38) = −2.04; p = .05
**AGE OF ONSET (years)**	22.94 (3.38)	16–28	–	–	–
**ILLNESS DURATION (years)**	11.25 (7.81)	2–24	–	–	–
ANTIPSYCHOTIC DOSE* (CPZ equivalent)	656.7 (385.4)	50–1200	–	–	–
**PANSS**					
*** Positive***	16.3 (5.7)	7–27	–	–	–
*** Negative***	16.1 (6.7)	7–30	–	–	–
*** General***	34.5 (11.7)	19–60	–	–	–
*** Total***	66.8 (20.1)	41–102	–	–	–
**WAIS-III IQ**					
*** Estimated full scale IQ***	93.9 (11.8)	75–115	105.8 (12.9)	84–128	t(38) = 3.0; p<.01
**HORMONE MEASUREMENTS**					
*** Testosterone (nmol/L)***	13.1 (5.4)	6.4–26	14.6 (5.6)	2.5–29.4	t(38) = −0.9; p>.10
*** Estrodial (pmol/L)***	139.1 (31.5)	88–192	136.3 (37.7)	<73–250	t(38) = 1.0; p>.10
*** Prolactin (mlU/ml)***	283.3 (287.4)	18–1130	165.4 (108.9)	64–581	t(38) = 1.6; p>.10
**DIAGNOSIS**	Schizophrenia				
	paranoid subtype n = 7				
	undifferentiated subtype n = 3				
	residual subtype n = 2				
	disorganized subtype n = 1				
	schizoaffective disorder				
	depressed subtype n = 4				
	bipolar subtype n = 1				

Means are listed, with standard deviations in parentheses.

### Hormone Assays

Serum testosterone, estradiol and prolactin levels did not differ between men with schizophrenia and healthy men (see Table 1). There were no strong significant correlations among hormone levels (testosterone, estradiol and prolactin), age, symptom severity or antipsychotic medication (CPZ equivalent daily dosage) in the men with schizophrenia. There was also no strong significant correlation between testosterone and prolactin level in either group (all p’s>.10, data not shown).

### Emotional Go/no-go Task Performance

A summary of behavioural results is presented in Table 2. Regarding RT, there was a significant main effect of condition, indicating slower reaction times in the *inhibit negative* condition (responding to neutral stimuli), F(1,38) = 48.1, p<.001, and a main effect of group, indicating that men with schizophrenia were slower overall, F(1,38) = 4.2, p<.05. The group by condition interaction for RT was not significant, F(1,38) = 1.3, p>.10. Regarding accuracy, there was a trend towards a significant main effect of condition, indicating decreased accuracy in the *inhibit negative* condition (responding to neutral stimuli), F(1,38) = 3.5, p = .07. A main effect of group indicated that men with schizophrenia were less accurate compared to healthy men, F(1,38) = 7.8, p<.01. The interaction between group and condition for accuracy was not significant, F<1. Error analysis revealed no significant main effect of condition or significant interaction for false alarms, both F<1. Men with schizophrenia tended to make more false alarm errors compared to healthy men, F(1,38) = 3.2, p = .08. There was trend towards a significant main effect of condition on the number of omissions, F(1,38) = 3.4, p = .07, with more omissions in the *inhibit negative* condition (responding to neutral stimuli). Men with schizophrenia made significantly more omission errors than the healthy men, F(1,38) = 9.1, p<.01. There was no significant interaction between group and condition, F<1. In men with schizophrenia, there was a strong trend for higher serum testosterone levels to be associated with greater accuracy in the *inhibit negative* condition (responding to neutral stimuli), r = .46, p = .06. There was no correlation between testosterone level and performance in the healthy men, r = .07, p>.10.

**Table 2:** Group means and standard deviations for behavioural performance parameters on the emotional Go/No-Go task.

	Patients		Controls	
	*Inhibit negative-* *respond to neutral*	*Inhibit Neutral- respond* *to negative*	*Inhibit negative-* *respond to neutral*	*Inhibit Neutral- respond* *to negative*
**RT [mean (SD), in ms]**	1029 (187)	890 (152)	967 (167)	774 (114)
**Accuracy [mean (SD), % correct]**	68 (15)	71 (16)	79 (13)	83 (16)
**False alarm errors [mean (SD), No. of errors]**	7 (6)	7 (4)	5 (4)	5 (5)
**Omissions [mean (SD), No. of errors]**	6 (4)	5 (5)	4 (2)	2 (3)

*Note: Reaction times refer to correct responses only. Accuracy reflects both responses to targets and withheld responses to distracters. False alarm errors indicate responses to distracters. Omissions indicate withheld responses to targets.*

### fMRI Results

#### Main task effects: inhibiting responses to negative versus neutral words

**Figure 1:**
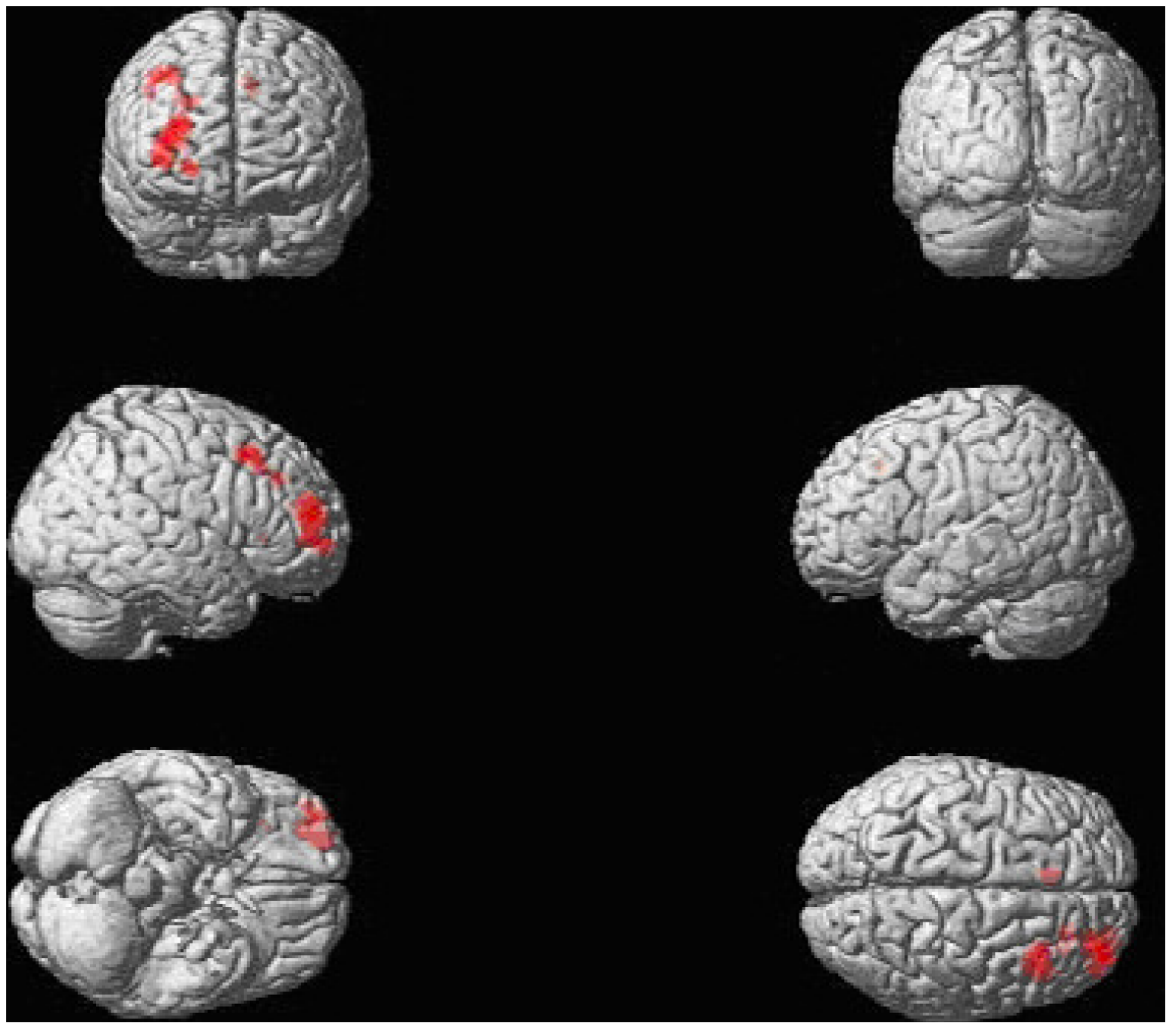
A whole-brain rendered image in the healthy men, showing significantly increased BOLD response when contrasting the inhibit negative and the inhibit neutral task conditions, p = .001 uncorrected with a minimum voxel extent k≥18. The healthy men showed a network of increased activation that overlapped with previous findings (Vercammen et al., 2012, *Journal of Psychiatry and Neuroscience,37(6): 379–388*). We applied the statistical criterion employed previously with this paradigm (Vercammen et al., 2012, *Journal of Psychiatric Research*), based on a double threshold approach. A simulation script was used to determine cluster threshold (cluster_threshold_beta.m retrieved from https://www2.bc.edu/~slotnics/scripts.htm), with the following parameters: acquisition matrix (80×80), original voxel dimensions (3×3×3), number of slices (32), full width half maximum (FWHM) set to 0, resampled voxel resolution (2×2×2), mask (none), corrected p-value (.05), voxel based p-value (.001), iterations (1000).

Table 3 presents an overview of the ROI analyses results. In the healthy males, we found a highly significant BOLD response increase in the right middle frontal gyrus ROI for the *inhibit negative versus neutral* contrast. Figure 1 displays a whole-brain rendered image for the contrast *inhibit negative versus neutral* in the healthy men, showing distinct activation of the middle frontal gyrus. A similar, but non-significant effect (after FDR correction) was observed in the left middle frontal gyrus ROI. In the men with schizophrenia, we observed a non-significant increase in BOLD response in the right middle frontal gyrus ROI (after FDR correction). Detailed whole brain results replicating previous findings [26] are presented in Table S1 and Figure S1.

**Table 3:** Results from the ROI analyses: (1) the main effect of the emotional go/no-go task, contrasting the inhibition of responses to negative stimuli with the inhibition of responses to neutral stimuli (“inhibit negative vs. neutral”) and (2) for the regression analysis of serum testosterone levels on BOLD response for the same contrast.

Effect/contrast	Group	Region-of-Interest	Hemisphere	Parameter estimate	T-value	P-value
Main task effect	Healthy men	**Middle frontal**	**Right**	**0.12**	**3.84**	0.000474*
for the contrast		**gyrus**	**Left**	**0.07**	**2.15**	**0.021658**
“inhibit		Insula	Right	0.05	0.94	0.178567
negative vs.			Left	−0.00	−0.00	0.501115
neutral”		Posterior	Right	−0.06	−0.91	0.812465
		cingulate	Left	−0.08	−1.19	0.876216
		Precuneus	Right	0.00	0.00	0.501371
			Left	−0.02	−0.36	0.637576
	Men with	**Middle frontal**	**Right**	**0.07**	**2.08**	**0.026604**
	schizophrenia	**gyrus**	**Left**	**0.07**	**2.08**	**0.026604**
		Insula	Right	0.03	0.67	0.256781
			Left	−0.02	−0.47	0.677971
		Posterior	Right	0.08	1.43	0.085147
		cingulate	Left	0.07	1.34	0.099512
		Precuneus	Right	0.06	1.12	0.138302
			Left	0.05	0.94	0.180713
Regression with	Healthy men	Middle frontal	Right	0.01	0.94	0.178352
testosterone as		gyrus	Left	0.00	0.67	0.254801
a predictor of		Insula	Right	0.00	−0.07	0.525823
BOLD response			Left	0.00	0.09	0.464033
for the contrast		Posterior	Right	−0.01	−0.75	0.770139
“inhibit		cingulate	Left	−0.01	−0.65	0.737152
negative vs.		Precuneus	Right	0.00	0.25	0.403758
neutral”			Left	0.00	0.12	0.450925
	Men with	**Middle frontal**	**Right**	**0.02**	**3.76**	0.000863*
	schizophrenia	**gyrus**	**Left**	**0.02**	**3.34**	0.002059*
		**Insula**	Right	0.01	1.11	0.141933
			**Left**	**0.02**	**2.37**	0.015376*
		Posterior	Right	0.00	0.11	0.457676
		cingulate	Left	0.00	0.28	0.392480
		**Precuneus**	Right	0.02	1.69	0.055591
			**Left**	**0.02**	**2.00**	**0.031633**

Separate analyses were conducted in the men with schizophrenia and the healthy men.

Note: *Indicates the effect survived FDR correction for multiple comparisons.

#### Association between testosterone level and task-related activation

**Figure 2:**
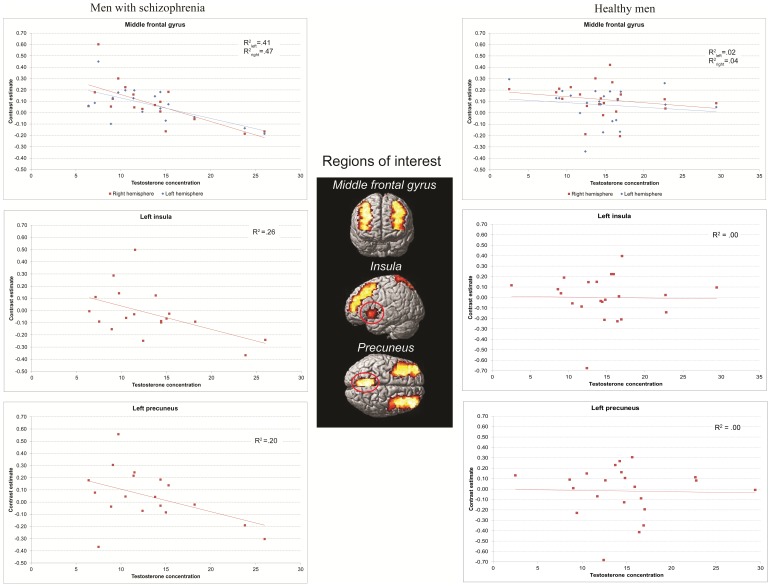
Results of a regression analysis in the men with schizophrenia and healthy men showing scatter plots of significant inverse correlations between testosterone levels and BOLD response in the bilateral middle frontal gyrus ROIs and the left insula ROIs for the inhibit negative versus neutral contrast in men with schizophrenia and no relationship in healthy men.

The regression analysis in the men with schizophrenia revealed a significant inverse correlation between testosterone levels and BOLD response in the bilateral middle frontal gyrus ROIs and the left insula ROIs for the *inhibit negative versus neutral* contrast (see Figure 2 and Table 3). A similar, but less robust correlation (not surviving FDR correction) was found for the left precuneus ROI. No significant associations were found for the remaining ROIs or the control region.

Although we found no correlation between serum prolactin and testosterone levels in either healthy men or men with schizophrenia (p>.10), due to the potential testosterone-inhibiting effect of prolactin, we ran additional analyses excluding participants with raised prolactin levels. Excluding the patient (n = 1) with relatively raised prolactin levels revealed findings consistent with our results reported above: a highly significant negative association between testosterone level and activation of the middle frontal gyrus ROIs (t = 3.08, p = .004 and t = 2.72, p = .008 for the right and left hemisphere, respectively), while the left insula and precuneus ROIs showed trend level effects (t = 1.59, p = .07 and t = 1.56, p = .07, respectively).

As estrogen effects have been demonstrated on neural responses during this task and it is known that estrogen and testosterone may interact, we conducted an additional analysis controlling for estrodial level. After adding estradiol as a nuisance covariate to the regression model with testosterone the negative association between serum testosterone level and activation of the middle frontal gyrus ROIs remained highly significant (t = 3.61, p = .001 and t = 3.03, p = .004, for the right and left hemisphere, respectively). The negative association between testosterone level and activation of the left insula ROI also remained significant (t = 2.15, p = .024), although it did not survive FDR correction.

There were no positive correlations between testosterone and brain activation in men with schizophrenia. None of the ROIs demonstrated an association (positive or negative) with serum testosterone level in healthy men.

#### Association among regional brain activation, task performance and clinical characteristics

In men with schizophrenia, there were strong, significant inverse correlations between task accuracy during the *inhibit negative* condition and activation of the right middle frontal gyrus ROI (r = −.51, p = .031) and the bilateral precuneus ROIs (r = −.50, p = .03 and r = −.47, p = .05). There were no significant correlations between brain activation and symptom severity or medication dose (all p’s>.10). In the healthy men, there were no strong, significant correlations with task performance. A detailed table of results is presented in Table S2.

## Discussion

Although there has been increasing interest in the role of sex steroids on emotion and cognition in neuropsychiatric disorders, to our knowledge this is the first study examining the relationship between circulating testosterone in men with schizophrenia and brain activation during a task integrating cognitive and emotional processing. We used an emotional go/no-go paradigm to investigate the neural substrate of emotion-cognition interaction, which is known to be affected in schizophrenia, as well as being sensitive to hormonal influences [25], [27]. Our study revealed an inverse association between serum testosterone and BOLD response of the bilateral middle frontal cortex and the left insula during inhibition of responses to negative emotional words in men with schizophrenia. There was no significant relationship between testosterone and brain activation in the healthy men. Interestingly, while higher testosterone levels were associated with reduced brain activity testosterone levels were also related to better task performance in men with schizophrenia, suggesting that increased normal levels of testosterone may have beneficial effects in terms of neural processing efficiency in men with schizophrenia. The absence of a difference in circulating testosterone and prolactin levels between men with schizophrenia and healthy men suggests that the observed effects of testosterone on the brain activation are specific to hormonal-neuronal interactions and they are not based on potential abnormalities in the available blood hormone levels. These results support and extend our recent findings [10] demonstrating that high-normal testosterone levels may be of cognitive benefit to men with schizophrenia and suggest that these cognitive benefits may occur through subtle changes in cortical processing.

### Emotional Go/no-go Performance and its Relationship to Testosterone Level

A consistent body of work has provided evidence of an interaction between “cold” cognitive and “hot” emotional processes. Affect can interfere with cognitive processes, including executive control and response inhibition [36], and conversely, executive control processes are used to regulate and manipulate the experience and expression of emotion [37]. The emotional go/no-go task assesses both the integrity of inhibition circuitry and potential perturbation by emotion processing, which is particularly relevant to disorders characterized by aberrant sensitivity to affective triggers such as schizophrenia [38]. Firstly, we found that across both participant groups the experimental manipulation of emotional content produced the expected behavioural effect: RT times were longer and accuracy was reduced when distracters were negative (inhibit responses to negative/respond to neutral stimuli). This can be explained by the fact that negative stimuli attract attention due to their increased salience, produce stronger response tendencies and retard inhibition, as has been observed in the emotional Stroop effect [39]. Secondly, men with schizophrenia showed an overall task performance deficit, suggesting a general impairment in response control, which is in agreement with recent reports [40]. Interestingly, in men with schizophrenia, performance in the *inhibit negative* condition tended to be enhanced in those with relatively higher endogenous testosterone. This finding appears to be in line with evidence from human and animal research implicating low testosterone in low mood and anxiety [41]–[44]. Attentional bias to negatively valanced information is associated with negative emotionality [45] and is common in neuropsychiatric disorders [46]. This bias tends to hamper the inhibition of responses to negative stimuli. Our findings suggest that higher endogenous testosterone levels may reduce this attentional bias to negative stimuli in schizophrenia, thereby facilitating the inhibition of automatic response tendencies to negative stimuli.

### Task-related Activation and its Relationship to Testosterone in Schizophrenia

Healthy men showed the expected pattern of activation: inhibiting responses to negative stimuli versus neutral stimuli was associated with robust increases in activation of the middle frontal gyrus ROI. On the other hand, men with schizophrenia demonstrated modest activation changes when inhibiting responses to negative versus neutral stimuli. The middle frontal gyrus has an established role in executive control and emotion/cognition interaction [47], [48]. Relatively reduced responsiveness in schizophrenia is in accord with previous findings from our lab [26]. Regression analysis revealed that BOLD response in the prefrontal ROI was significantly and inversely associated with serum testosterone level in men with schizophrenia, but not in healthy men. An inverse relationship with testosterone was also observed in the left insula and precuneus, which play a part in self-referential and interoceptive processing and its integration with perceived emotional salience [49], [50]. Our findings are thus consistent with a modulatory impact of sex steroid signalling on the neural substrates of cognitive control in the context of emotion processing in schizophrenia. The direction of the association indicated that at higher normal levels of circulating testosterone, BOLD response was relatively reduced while inhibiting responses to negative stimuli. Interestingly, improved performance on the *inhibit negative* task condition tended to be associated with higher testosterone as well as reduced activation of the testosterone-sensitive regions (middle frontal gyrus and precuneus). These associations may suggest that – under certain circumstances – testosterone can produce more efficient processing in integral components of the neural network subserving emotion-guided actions and produce more adaptive behavioural output. At first glance, this finding may seem contradictory to the observation of reduced task-related activation in men with schizophrenia, as reduced activity has previously been interpreted as evidence of deficient processing in schizophrenia. We propose that when testosterone level is not taken into account, inter-individual variability in men with schizophrenia may result in less consistent activation patterns when assessed at the group level. However, within the group of men with schizophrenia, slight variations within the normal range of testosterone levels do appear to account for some of the systematic variability in the neural response to these specific task demands. These findings provide novel intriguing evidence of the possibility that sex steroids may be important modulators of task-related brain function in schizophrenia. More work is required to fully clarify the exact relationship between brain function, behavioural responses and endogenous hormones and the degree to which activation patterns associated with hormonal variation are in fact “abnormal”.

Our data indicate that the relationship between sex steroids and brain activation differs between men with schizophrenia and healthy men and that key structures dedicated to attentional direction and cognitive control over emotion processing are involved. There is a possibility that a more complex, non-linear relationship exists in healthy men. However, the group differences observed in this study (Figure 1) suggest that within the normal range, small variations in testosterone levels do not greatly impact emotional and cognitive processing in healthy men, whereas normal variation in testosterone levels can influence brain activity and behaviour in schizophrenia.

### Limitations and Future Directions

The need to apply a strong, hypothesis driven approach to detect a significant relationship between testosterone and brain activity in specific regions may reflect the fact that the effect size of the comparison between two task conditions that differ only in terms of the emotional valance of the stimuli appear to be relatively small. The use of antipsychotics in the men with schizophrenia must be considered carefully as a potential confound as prolactin-elevating antipsychotics have been shown to influence testosterone levels [51]. However, in our sample, only 6 men with schizophrenia were receiving prolactin raising antipsychotics and the overall prolactin level did not differ significantly between schizophrenia and control groups. While many other factors appear to influence circulating testosterone levels in males (e.g., ethnicity, BMI, smoking, marital status, fatherhood, seasonality, and in men with schizophrenia: antipsychotics), our samples were well matched on age and ethnicity and we found no significant group differences in testosterone or prolactin levels, suggesting that these variables are unlikely to account for our findings.

We measured circulating total testosterone which is an indirect measure of free testosterone. Most circulating testosterone is bound to sex hormone binding protein which leaves only a small proportion to enter the cells and bind to receptors. However, free testosterone correlates well with total testosterone and both have been used as an index of bioavailable testosterone [52]. These novel findings open up an avenue for further research to explore the extent of testosterone-induced modulation of the neural response during cognitive and/or affective challenges in schizophrenia. Future studies may consider assessing the impact of sex hormone binding globulin and other circulating hormones such as corticosteroid, thyroid hormones and estrogen on brain activity in schizophrenia.

## Conclusion

To our knowledge the current study is the first to link circulating testosterone levels to functional neural correlates of cognitive-emotional processing in men with schizophrenia. Within the context of normal-range circulating hormone levels, we observed an inverse relationship between testosterone and fMRI BOLD response in task-specific prefrontal brain regions during emotion-guided response inhibition. The fact that higher endogenous testosterone was associated with improved behavioural performance and a reduction in brain activation, suggests a role of sex steroid signalling in enhancing neural efficiency during negative emotional processing in schizophrenia.

## Supporting Information

Figure S1 Thresholded T-map on sagittal sections, for the contrast *inhibit negative>inhibit neutral*, with a less conservative p-value = .01. Regions showing increased activation in the healthy men are indicated in yellow, regions showing increased activation in the men with schizophrenia are indicated in red. The figure demonstrates that there is some overlap in activation between the two groups, but that the pattern is less robust and shows a less contiguous spatial distribution in the men with schizophrenia as compared to the healthy men.
(TIF)

Table S1 Overview of clusters showing significantly increased BOLD response when contrasting the *inhibit negative* and the *inhibit neutral* task conditions, p = .001 uncorrected; min. voxel extent k ≥18. We chose to apply the same statistical criterion as we employed in an earlier publication on this paradigm (Vercammen et al., 2012, Journal of Psychiatric Research), based on a double threshold approach. A simulation script was used to determine cluster threshold (cluster_threshold_beta.m retrieved from https://www2.bc.edu/~slotnics/scripts.htm), with the following parameters acquisition matrix (80×80), original voxel dimensions (3×3×3), number of slices (32), full width half maximum (FWHM) set to 0, resampled voxel resolution (2×2×2), mask (none), corrected p-value (.05), voxel based p-value (.001), iterations (1000). The healthy men showed a network of increased activation in the *inhibit negative* condition compared to the *inhibit neutral condition*, that overlapped with previous findings from our group (Vercammen et al., 2012, *Journal of Psychiatry and Neuroscience,37(6): 379–388*). The men with schizophrenia did not show significant activation changes at the same significance level. Lowering the statistical threshold did reveal a number of activation clusters in the patient group.
(DOC)

Table S2 A table displaying the correlations between activation of the bilateral ROIs for the contrast *inhibit negative>inhibit neutral*, and clinical characteristics in the sample of men with schizophrenia. Significant correlations are indicated in bold.
(DOCX)
