# Supplementary figures and images for: Testosterone Is Inversely Related to Brain Activity during Emotional Inhibition in Schizophrenia
Source: PLoS One. 2013 Oct 31;8(10):e77496. doi: 10.1371/journal.pone.0077496 (PMC3814976; doi:10.1371/journal.pone.0077496)

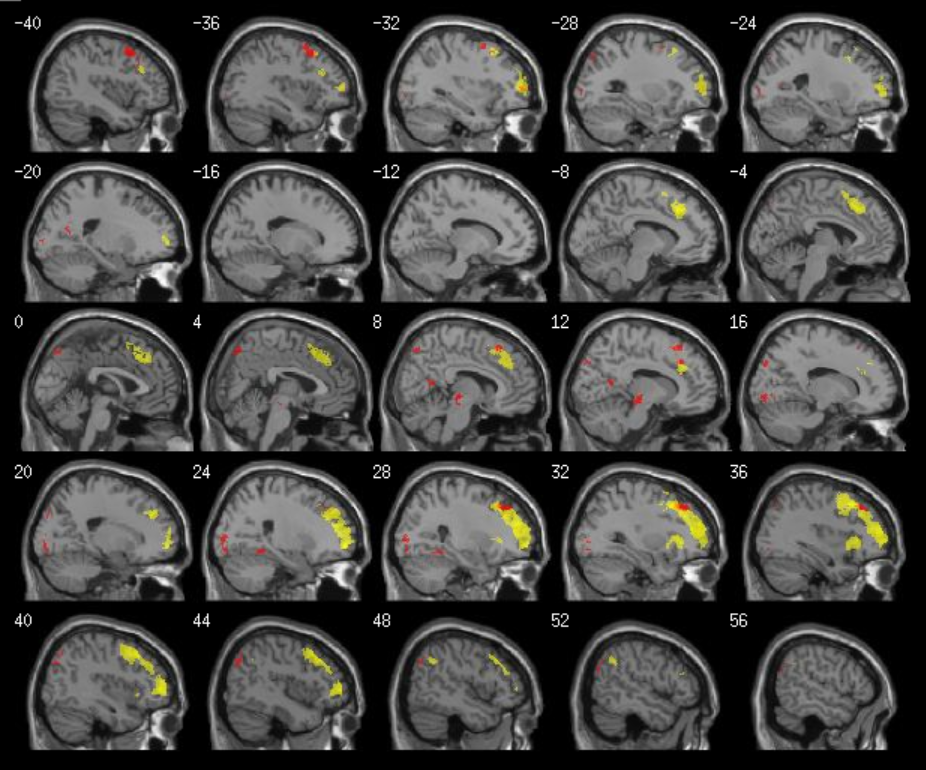

Supplement: Figure S1 — Thresholded T-map on sagittal sections, for the contrast inhibit negative>inhibit neutral, with a less conservative p-value = .01. Regions showing increased activation in the healthy men are indicated in yellow, regions showing increased activation in the men with schizophrenia are indicated in red. The figure demonstrates that there is some overlap in activation between the two groups, but that the pattern is less robust and shows a less contiguous spatial distribution in the men with schizophrenia as compared to the healthy men. (TIF) [file pone.0077496.s001.tif]
