# Supplementary material for: Testosterone Is Inversely Related to Brain Activity during Emotional Inhibition in Schizophrenia
Source: PLoS One. 2013 Oct 31;8(10):e77496. doi: 10.1371/journal.pone.0077496 (PMC3814976; doi:10.1371/journal.pone.0077496)
Supplement: Table S1 — Overview of clusters showing significantly increased BOLD response when contrasting the inhibit negative and the inhibit neutral task conditions, p = .001 uncorrected; min. voxel extent k ≥18. We chose to apply the same statistical criterion as we employed in an earlier publication on this paradigm (Vercammen et al., 2012, Journal of Psychiatric Research), based on a double threshold approach. A simulation script was used to determine cluster threshold (cluster_threshold_beta.m retrieved from https://www2.bc.edu/~slotnics/scripts.htm), with the following parameters acquisition matrix (80×80), original voxel dimensions (3×3×3), number of slices (32), full width half maximum (FWHM) set to 0, resampled voxel resolution (2×2×2), mask (none), corrected p-value (.05), voxel based p-value (.001), iterations (1000). The healthy men showed a network of increased activation in the inhibit negative condition compared to the inhibit neutral condition, that overlapped with previous findings from our group (Vercammen et al., 2012, Journal of Psychiatry and Neuroscience,37(6): 379–388). The men with schizophrenia did not show significant activation changes at the same significance level. Lowering the statistical threshold did reveal a number of activation clusters in the patient group. (DOC) [file pone.0077496.s002.doc]

| Contrast |  | Peak voxel coordinate | | | Intensity (T-value) | Number of voxels | Region |
| --- | --- | --- | --- | --- | --- | --- | --- |
|  |  | x | y | z |  |  |  |
| Inhibit negative | Healthy men | 24 | 58 | -4 | 4.92 | 53 | Right superior orbitofrontal gyrus |
| > inhibit neutral |  | 34 | 28 | 0 | 3.89 | 18 | Right insula |
|  |  | 36 | 52 | 12 | 4.51 | 356 | Right middle frontal gyrus (BA10) |
|  |  | 30 | 34 | 34 | 4.28 | 54 | Right middle frontal gyrus (BA9) |
|  |  | 34 | 18 | 46 | 5.01 | 154 | Right middle frontal gyrus (BA8) |
|  |  | -8 | 26 | 40 | 4.87 | 65 | Left superior medial frontal gyrus / cingulate |
|  | Men with schizophrenia | *No significant clusters* | | | | | |
| Inhibit neutral | Healthy men | -24 | -10 | -14 | 4.50 | 62 | Left parahippocampal gyrus |
| > inhibit negative | Men with schizophrenia | *No significant clusters* | | | | | |
