# Supplementary material for: Testosterone Is Inversely Related to Brain Activity during Emotional Inhibition in Schizophrenia
Source: PLoS One. 2013 Oct 31;8(10):e77496. doi: 10.1371/journal.pone.0077496 (PMC3814976; doi:10.1371/journal.pone.0077496)
Supplement: Table S2 — A table displaying the correlations between activation of the bilateral ROIs for the contrast inhibit negative>inhibit neutral, and clinical characteristics in the sample of men with schizophrenia. Significant correlations are indicated in bold. (DOCX) [file pone.0077496.s003.docx]

TABLE S2

|  | | Chlorpromazine equivalent dose | | Serum Prolactin | | PANSS - positive | | PANSS - negative | | PANSS - general | | % correct on *inhibit negative* task condition | |
| --- | --- | --- | --- | --- | --- | --- | --- | --- | --- | --- | --- | --- | --- |
|  | | r | p | r | p | r | p | r | p | r | p | r | p |
| Posterior cingulate | Left | 0.19 | 0.46 | 0.10 | 0.71 | -0.37 | 0.13 | -0.01 | 0.96 | -0.20 | 0.42 | -0.33 | 0.18 |
|  | Right | 0.23 | 0.36 | 0.12 | 0.65 | -0.32 | 0.20 | -0.06 | 0.82 | -0.10 | 0.69 | -0.29 | 0.24 |
| Middle frontal gyrus | Left | -0.16 | 0.53 | -0.22 | 0.39 | -0.05 | 0.84 | 0.01 | 0.96 | -0.11 | 0.67 | -0.45 | 0.06 |
|  | Right | -0.17 | 0.51 | -0.21 | 0.41 | -0.02 | 0.95 | -0.19 | 0.46 | -0.26 | 0.30 | **-0.51** | **0.03** |
| Insula | Left | 0.14 | 0.58 | -0.16 | 0.54 | 0.25 | 0.31 | 0.05 | 0.84 | 0.26 | 0.30 | -0.30 | 0.23 |
|  | Right | 0.05 | 0.84 | -0.01 | 0.95 | 0.06 | 0.83 | -0.13 | 0.60 | 0.07 | 0.78 | -0.16 | 0.52 |
| Precuneus | Left | 0.36 | 0.15 | -0.16 | 0.53 | -0.09 | 0.72 | 0.20 | 0.43 | 0.09 | 0.73 | **-0.50** | **0.03** |
|  | Right | 0.38 | 0.12 | -0.14 | 0.57 | -0.05 | 0.84 | 0.18 | 0.47 | 0.14 | 0.59 | **-0.47** | **0.05** |
